# A survey of East Mediterranean *Dasumia* (Araneae, Dysderidae) with description of new species

**DOI:** 10.3897/zookeys.137.1783

**Published:** 2011-10-14

**Authors:** Kadir Boğaç Kunt, Recep Sulhi Özkütük, Mert Elverici

**Affiliations:** 1 Poligon Sitesi 71/27-B TR-06810 Dodurga, Çayyolu, Ankara, Turkey; 2 Department of Biology, Faculty of Science, Anadolu University, TR- 26470 Eskişehir, Turkey; 3 Department of Biological Sciences, Faculty of Arts and Sciences, Middle East Technical University, TR-06531 Ankara, Turkey

**Keywords:** Harpacteinae, spider, Turkey

## Abstract

*Dasumia gasparoi* **sp. n.** isdescribed based on specimens of both sexes. The new species is compared with the similar *Dasumia crassipalpis* (Simon, 1882), described from Syria; and with *Dasumia mariandyna* Brignoli, 1979, the only previously known species of the genusrecordedfrom Turkey. Furthermore, we point out that, due to some contradictions to the original description of the genus, *Dasumia mariandyna* may necessarily belong to another genus. Detailed morphological descriptions, diagnosis and figures of the copulatory organs of the new species are presented.

## Introduction

*Dasumia* is a genus of the family Dysderidae and includes 13 previously described species (Platnick 2011). Ten are distributed in Europe, two in the Middle East and one in Turkey. *Dasumia* belongs in the subfamily Harpacteinae, and differs from other genera by the absence of ventral spines on the metatarsi and anterior tibiae; the posterior tarsi with either two claws or with an additional single tiny claw; by the typical arrangement of the cheliceral dentition and by having an abruptly curled embolus in males or more or less sclerotized posterior diverticulum of vulva in females (Thorell 1875; Dunin 1992; Deeleman-Reinhold 1993).

During our survey of the Turkish spider fauna, we encountered some interesting dysderid specimens in Kahramanmaraş province, a region that constitutes a transition zone between the Turkish Mediterranean region and the south-eastern region of Anatolia. Initially, examination of the sternum morphology suggested the specimens were members of the subfamily Harpacteinae. However, the structure of copulatory organs did not conform with the known species of *Harpactea* Bristowe, 1939 and *Stalagtia* Kratochvíl, 1970 from Turkey, nor did they show any similarity with those of *Dasumia mariandyna* Brignoli, 1979, which represented the only known *Dasumia* species recorded from Turkey. Kahramanmaraş is located close to Syria, so we then examined members of Harpacteinae known from Syria and the Middle East. This revealed similarities between our specimens and those of *Dasumia crassipalpis* from Syria, which had previously been described as *Harpactes crassipalpis* by Simon (1882) and later transferred to *Dasumia* by Alicata (1974),based on the structure of the previously unknown female genitalia.

The purpose of this study is to describe and illustrate a new species of *Dasumia* from Turkey and to discuss its placement in the genus together with the Syrian *Dasumia crassipalpis* and the Turkish endemic *Dasumia mariandyna*.

## Materials and methods

**Figure 1.**
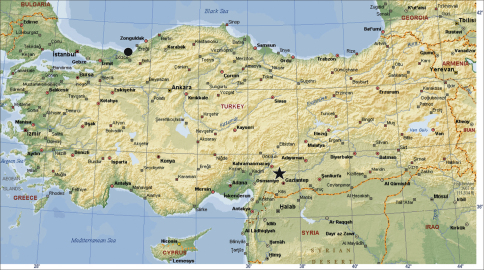
Collecting localities of Turkish *Dasumia* species. ★ terra typica, *Dasumia gasparoi* sp. n. **●** *Dasumia mariandyna*

All specimens were collected from Kahramanmaraş province of Turkey (Fig. 1). The specimens were collected from under stones using a hand aspirator. Digital images of the pedipalps and vulvae were taken with a Leica DFC295 digital camera attached to a Leica S8AP0 stereomicroscope, with 5–15 photographs taken in different focal planes and combined using image stacking software. Photographic images were edited using PHOTOSHOP CS2 and COREL-DRAW X3 was used to create the plates. All measurements are in mm. Terminology for the body measurements follows Chatzaki and Arnedo (2006). Terminology for the copulatory organs is adapted from Alicata (1974) and Deeleman-Reinhold (1993). On the male copulatory organ, additional apophyses developed on the structure called the “Apophysis_a_" are named as “Apophysis_a1,_ _a2_, etc" relating to their sequential order relative to that of Apophysis_a1._ The following abbreviations are used in the text: **AL**, abdominal length; **CL**, carapace length; **CWmax**, maximum carapace width; **CWmin**, minimum carapace width; **AME**, anterior median eyes; **PLE**, posterior lateral eyes; **PME**, posterior median eyes; **AMEd**, diameter of anterior median eyes; **PLEd**, diameter of posterior lateral eyes; **PMEd**, diameter of posterior median eyes; **ChF**, length of cheliceral fang; **ChG**, length of cheliceral groove; **ChL**, total length of chelicera (lateral external view); **Ta**, tarsus; **Me**, metatarsus, **Ti**, tibia; **Pa**, patella; **Fe**, femur; **Tr**, trochanter; **C**, coxa; **D**, dorsal; **pl**, prolateral; **rl**, retrolateral; **V**, ventral; **cKBK**, Personal collection of Kadir Boğaç Kunt, Ankara, Turkey; **AUZM**, Anadolu University, Zoology Museum, Eskişehir, Turkey; **SMF**, Senckenberg Museum, Frankfurt am Main, Germany.

## Taxonomy

***Dasumia* Thorell, 1875**

**In Thorell, 1875: 100, type species** ***Dasumia taeniifera* Thorell, 1875**

### Dasumia gasparoi sp. n.

urn:lsid:zoobank.org:act:8E19F1DC-74BA-47D4-A505-6498414B4CCE

http://species-id.net/wiki/Dasumia_gasparoi

#### Material examined.

 **Holotype.** ♂ (AUZM), TURKEY, **Kahramanmaraş Province**, Pazarcık District, c. 5 km S of Narlı Town [37°19'11.78"N; 37°10'16.19"E], 07.03.2008, under stones, leg. E.A.Yağmur. **Paratypes:** 1 ♀ (AUZM); 1 ♀ (SMF), together with holotype.

#### Derivatio nominis.

The new species is named in honour of the Italian geologist & arachnologist Dr. Fulvio Gasparo, who has made great contributions to the taxonomy of the family Dysderidae.

#### Diagnosis.

*Dasumia gasparoi* sp. n. can be readily identified by the unique structure of male and female copulatory organs. It is most similar to *Dasumia crassipalpis* from which it can be differentiated as follows:

1. In *Dasumia gasparoi* sp. n. the transition zone between the tegulum and the distal appendages is more notable than in *Dasumia crassipalpis*.

2. In *Dasumia gasparoi* sp. n.the tip of the falciform embolus is sharper and taller and the embolus extends beyond Apophysis_b_, whereas in *Dasumia crassipalpis*, the embolus only reaches the middle of Apophysis_b_.

3. Apophysis_a_ and Apophysis_b_ show explicit differences in structure between the two species.

4. In *Dasumia gasparoi* sp. n. the spermatheca are relatively wider. Distal crest of spermatheca is shorter and thicker in *Dasumia gasparoi* sp. n. than in *Dasumia crassipalpis* (see Alicata 1974).

#### Measurements.

(Holotype ♂ / Paratype n=2 ♀)**: AL** 3.50 / 4.47-4.50; **CL** 3.20 / 3.25-3.50; **CWmax** 2.50 / 2.75-2.80; **CWmin** 1.25 / 1.59-1.44 ; **AMEd** 0.16 / 0.17-0.18; **PLEd** 0.15 / 0.14-0.15; **PMEd** 0.11 / 0.14-0.12 ; **ChF** 0.58 / 0.66-0.66; **ChG** 0.47 / 0.52-0.53 ; **ChL** 1.37 / 1.60-1.62. Leg measurements are given in Table 1.

**Table 1.** Leg measurements of *Dasumia gasparoi* sp. n.

**(Holotype** ♂ **/ Paratype** ♀**)**	**Fe**	**Pa**	**Ti**	**Me**	**Ta**
**Leg I**	3.00 / 3.08	1.80 / 1.88	2.76 / 2.60	2.68 / 2.48	0.63 / 0.48
**Leg II**	2.50 / 2.56	1.60 / 1.68	2.40 / 2.24	2.60 / 2.50	0.63 / 0.60
**Leg III**	2.10 / 2.16	1.05 / 1.12	1.75 / 1.68	2.05 / 2.04	0.55 / 0.44
**Leg IV**	2.96 / 3.00	1.40 / 1.60	2.50 / 2.56	2.64 / 3.20	0.63 / 0.64

#### Description.

**Figures 2–5.**
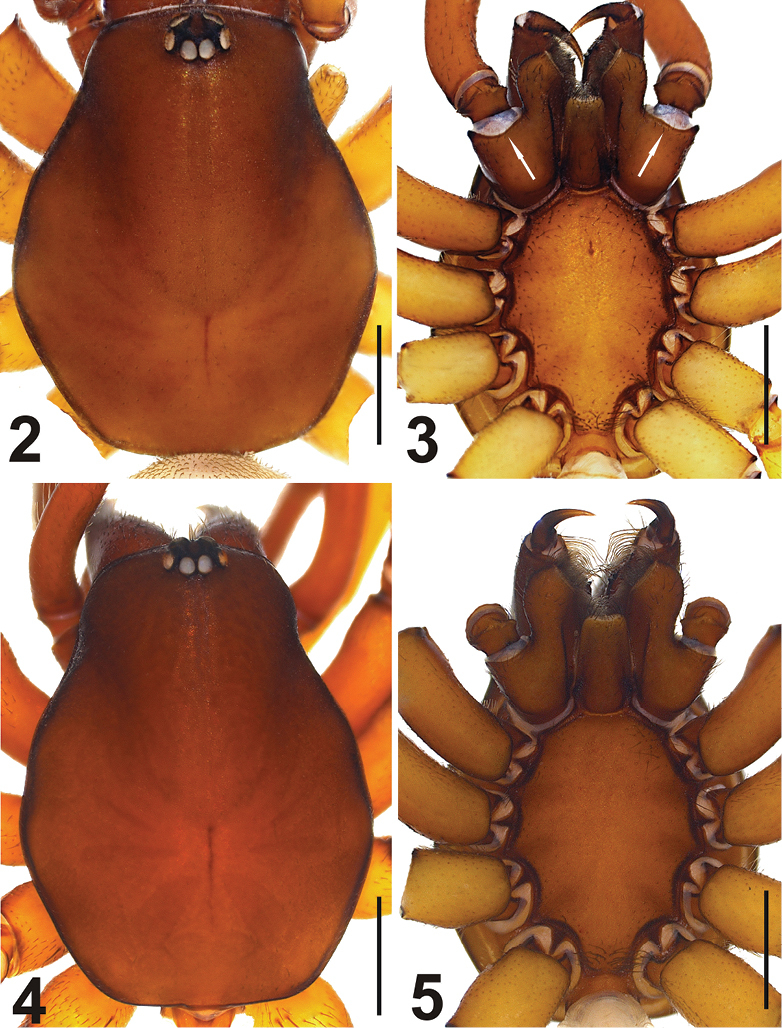
*Dasumia gasparoi* sp. n. **2**, **3** (♂) carapace, sternum **4**, **5** (♀) ditto. Scale lines: 0.25 mm.

**Figures 6–8.**
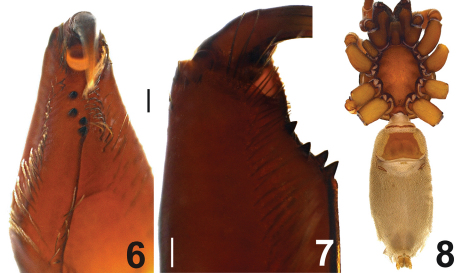
*Dasumia gasparoi* sp. n. **6, 7** cheliceral teeth **8** female, ventral view Scale line: (6, 7) 0.1 mm.

Carapace dark brown anteriorly, yellowish brown posteriorly and blackish brown laterally. AME, PLE and PME in a circular arrangement. AME separated. PLE and PME clearly separated. Sternum, labium, gnathocoxae and chelicerae yellowish brown. Sternum blackish brown laterally (Figs 2–5). Cheliceral groove with two retromarginal and two promarginal teeth. Teeth on the promargin originate at the base of the groove and end in the middle. Retromarginal teeth originate in alignment with the point at which the promarginal teeth stop, and continue to the top of the cheliceral groove. Teeth on retromargin relatively smaller and more widely separated, when compared with those on the promargin (Figs 6, 7). Cheliceral groove long, top of the labium and gnathocoxae covered with short hairs. In males, joint of trochanter to gnathocoxa thicker and deeper (see Fig. 3). Abdomen greyish to light brown, with short, thin blackish hair over the entire surface. Females with a strongly developed linear postpedicelar and trapezoid epigastric scutum (Fig. 8). Males also have these structures, but they appear thinner and have less colour. Legs yellowish to light brown with sparse blackish setae. Periphery of articulation points dark brown.

**Figures 9–12.**
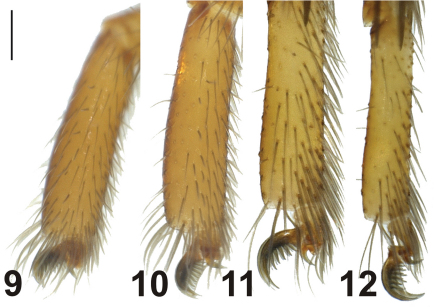
Leg tarsi of *Dasumia gasparoi* sp. n. **9** Leg I **10** Leg II **11** Leg 3 **12** Leg IV Scale line: 0.25 mm.

Leg IV > Leg I > Leg II > Leg III. Tarsi with three claws. Bent claws and middle claws are well developed (Figs 9, 10, 11, 12).

Tarsi III and IV with fine scopulae (Figs 9-12). Legs III and IV with fine metatarsal scopulae along the ventral surface, covering slightly less than the distal half of the segment. Dorsal part of coxae III and IV with 1-4 spines. Details of leg spination are given in Table 2.

**Figures 13–16.**
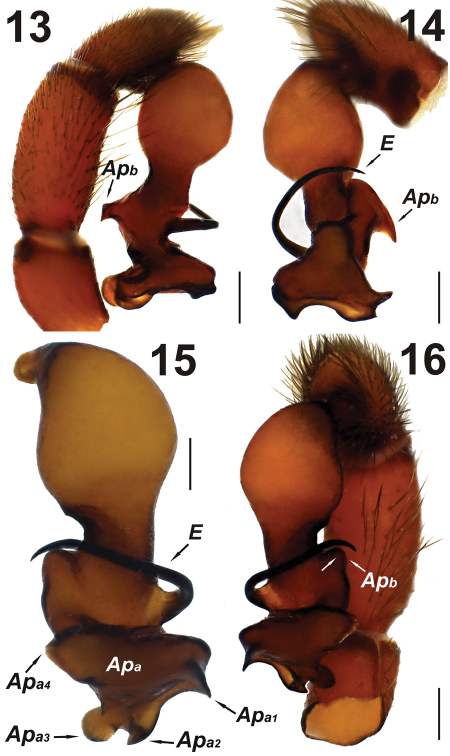
Male palp of *Dasumia gasparoi* sp. n. Abbreviations: ***Ap_a_***Apophysis_a _***Ap_b_*** Apophysis_b_ ***E*** embolus. Scale lines: 0.25 mm.

In males, palpal tibia almost double the size of the tarsus. Tarsus bullet-shaped in lateral view. Tegulum yellowish brown; approximately as long as wide, and with a spherical shape. Between the distal appendages and tegulum, there is a visible transition region, peripherally sclerotized in places (Figs 13, 14). Tip of embolus adjacent to Apophysis_b_ (Figs 13, 15). Embolic base wide and triangular. Embolus falciform, tapering distally, blackish and well sclerotized along its length (Figs 15, 16). Apophysis_a_ triangular, separated from embolus and Apophysis_b_ (Fig. 13). Details of palp in ventral view: Apophysis_a1_ short and sharp, beak-shaped at the right corner; Apophysis_a2_ semicircular at the left corner; Apophysis_a3 _(which is stubbier than apophyses_a1_ and Apophysis_a4_)ear-shaped at the rear corner. All of these apophyses with well sclerotized margins (Fig. 15).

**Figures 17–20.**
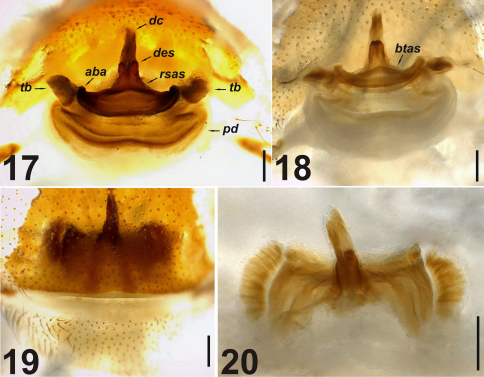
Vulva of *Dasumia gasparoi* sp. n. **17, 18** dorsal view **19, 20** ventral view. Abbreviations: ***aba*** anterior basal arc ***btas*** basal transverse part of the anterior spermatheca ***dc*** distal crest ***des*** distal expansion of the spermatheca ***pd*** posterior diverticulum ***rsas*** rod-shaped part of the anterior spermatheca ***tb*** transverse bar. Scale lines: 0.5 mm.

Vulva generally well sclerotized. Distal crest medium-sized and butt-ended. Distal expansion of the spermatheca wider than distal crest and visually hump-shaped. Rod-shaped part of the anterior spermatheca short and broader towards the base. Basal transverse part of the anterior spermatheca appears merged with the anterior basal arc. Both structures well sclerotized from centre to periphery. In dorsal view, anterior basal arc arc-shaped; basal transverse part of the anterior spermatheca forming a downward chevron shape. Transverse bar longer than the anterior basal arc. The surface area of the posterior spermatheca is wider than the anterior spermatheca. Transverse bar ends with one snake head-shaped structure at either side; and in contact with posterior diverticulum over complex membranous channel network (Figs 17, 18, 19).

**Figures 24–25.**
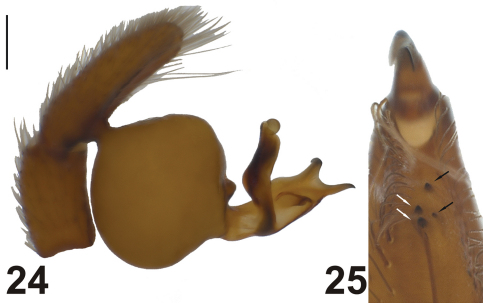
*Dasumia mariandyna* (topotype). **24** male palp **25** cheliceral teeth. Scale line: (24) 0.25 mm.

#### Note.

 In ventral view, and looking at an angle of 70° from the surface to the vulva, we observed symmetrically located, reniform structures consisting of helicoidal canals inside both sides of the vulva (Fig. 20). The origin and function of these structures is unknown.

#### Ecology.

 Samples were collected during early spring from under stones (using a hand aspirator) in steppe habitat with scrubs of *Quercus coccifera* and with pine woods located close by. The collection locality was on low land at the middle of a mountainous region, which may enhance the probability of this species being an endemic.

### Dasumia crassipalpis

(Simon, 1882)

http://species-id.net/wiki/Dasumia_crassipalpis

Harpactes crassipalpis : Simon, 1882: 224, f. 7-8 (D ♂).
Harpactocrates crassipalpis : Reimoser, 1919: 11.
Dasumia crassipalpis : Alicata, 1974: 40, f. 1-4 (T ♂ from *Harpactocrates*, D ♀).

#### Material examined. 

1 ♂ (AUZM), **ISRAEL**, Mount Meron, 17.XII.2010, leg. C. Drees

#### Detailed comparison of Dasumia gasparoisp. n. andDasumia crassipalpis.

 Unfortunately, due to lack of material, we were unable to compare females of the two species. Here we comment on general similarities and differences observed from comparison of male specimens from both species; and from the description of female *Dasumia crassipalpis* given by Alicata (1974) with the female of *Dasumia gasparoi* sp. n., as follows:

Body coloration and general appearance similar in both species.

Arrangement of cheliceral teeth on cheliceral groove similar, but in *Dasumia crassipalpis*, distance between teeth on promargin and retromargin relatively wider.

In the original description of *Dasumia crassipalpis*, carapace width for males was given as 3.2 mm (see Simon 1882, page 224). Our *Dasumia crassipalpis* specimen from Israel has a carapace width of 3.26 mm. Based on the body measurements of *Dasumia gasparoi* sp. n., there are no significant differences between the two species. However, the legs of *Dasumia crassipalpis* from Israel are relatively shorter than *Dasumia gasparoi* sp. n. (see Table 3).

Leg spination similar in both species. Legs III and IV of female *Dasumia gasparoi* sp. n. and leg IV of male *Dasumia crassipalpis* exhibit trochanteric retrolateral spines, which is an interesting observation (see Table 2 and 4).

Linear postpedicelar and trapezoid epigastric scutum present in males of both species, in *Dasumia gasparoi* sp. n. pale; in *Dasumia crassipalpis* even paler.

**Table 2.** Leg spination of *Dasumia gasparoi* sp. n.

♂ **(Holotype)**	**Leg I**	**Leg II**	**Leg III**	**Leg IV**
**C**	0	0	2 pl	3 pl 1 D
**Tr**	0	0	0	0
**Fe**	4 pl	5 pl	3 D 4 rl	9 D
**Pa**	0	0	2 D 1 rl	0
**Ti**	0	0	2 pl 1 D 4 rl 5 V	4 pl 4 rl 5 V
**Me**	0	0	3 pl 6 rl 2 V	4 pl 1 D 5 rl 6 V
♀ **(Paratype)**				
**C**	0	0	1 pl	2 pl
**Tr**	0	0	1 rl	1 rl
**Fe**	2 pl	1 pl	3 D 3 rl	8 D
**Pa**	0	0	2 D 1 rl	0
**Ti**	0	0	2 pl 1 D 3 rl 2 V	4 pl 1 D 3 rl 5 V
**Me**	0	0	4 pl 6 rl 2 V	4 pl 4 rl 5 V

**Table 3.** Leg measurements of *Dasumia crassipalpis*****

♂	**Fe**	**Pa**	**Ti**	**Me**	**Ta**
**Leg I**	2.67	1.85	2.65	2.57	0.64
**Leg II**	2.69	1.66	2.54	2.49	0.62
**Leg III**	2.20	1.16	1.75	2.24	0.57
**Leg IV**	3.12	1.49	2.60	3.06	0.58

**Figures 21–23.**
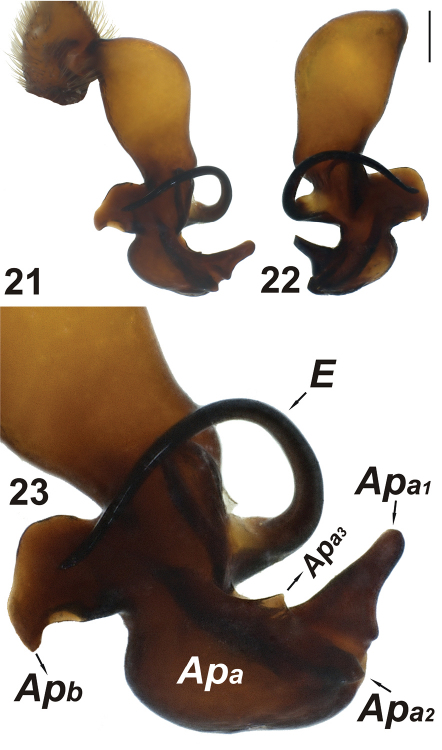
Male palp of *Dasumia crassipalpis*. Abbreviations: ***Ap_a_***Apophysis_a _***Ap_b_*** Apophysis_b_ ***E*** embolus. Scale line (21, 22): 0.25 mm.

In *Dasumia crassipalpis*, morphology of the distal appendages distinctive on male palp. Apophysis_b_ longer and wider. Also, in *Dasumia gasparoi* sp. n., Apophysis_a1_ shorter and projecting downwards; while in *Dasumia crassipalpis* it is well developed, apparent and projected upwards. In *Dasumia crassipalpis* palp when viewed ventrally, except for Apophysis_a1_, the remaining apophyses are located at the right corner of Apophysis_a_, close to Apophysis_a1 _(Figs 21, 22, 23).

Even though there are structural differences apparent, the vulvae of both species are similar and generally well sclerotized (see Alicata 1974).

#### A short assessment on the distribution of 

*Dasumia crassipalpis*.First described as *Harpactes crassipalpis* by Simon (1882) on the basis of male specimens collected from Syria, females were subsequently described by Alicata (1974); and based on the previously unknown female genitalia, the species was transferred to the genus *Dasumia*. Simon reported the terra typica of *Dasumia crassipalpis* as “Syria", without giving any further detail (see Simon 1882). Moreover, Syria was the land of the Ottoman Empire in those days, and some provinces today known as Turkish were included with the rest of the region then known as Syria. For this reason, it is hard to draw a northern border line for the distribution of the species. However, considering that the only male specimen examined for the purpose of this study was collected in Israel, it may be assumed that *Dasumia crassipalpis* is distributed along the line of Syria, Lebanon and Israel. Although we have not yet collected this species during our extensive arachnological field studies at the Turkey-Syria border, it is possible that this species reaches Turkey at the north and Jordan at the south of its range.

### Dasumia mariandyna

Brignoli, 1979

http://species-id.net/wiki/Dasumia_mariandyna

Dasumia mariandyna : Brignoli, 1979: 312, f. 9-11 (D ♂♀).
Dasumia mariandyna : Le Peru, 2011: 222, f. 240 (♂♀).

#### Material examined.

 1 ♂ (AUZM) **TURKEY**, **Düzce Province**, Akçakoca District, c. 1 km south of Kepenç Village [41°4'11.89"N; 31°7'9.06"E], 22.V.2008, under leaf litter, leg. K.B. Kunt; 1 ♂ (AUZM), **Bolu Province**, Abant [40°40'39.36"N; 31°28'18.78"E], 13.IX.2009, under leaf litter, leg. K.B. Kunt.

#### Comparison of Dasumia gasparoi sp. n. and Dasumia mariandyna. 

Brignoli defined the relation of *Dasumia mariandyna* to other species of the genus as follows: “The new species is not related to the Greek and Near Eastern species; it can be easily distinguished from all known species by the genitalia" (see Brignoli 1979, page 313). Indeed, *Dasumia mariandyna* can be easily distinguished by the copulatory organs from the Middle Eastern representative of the genus, *Dasumia crassipalpis* and from *Dasumia gasparoi* sp. n. which is very close to *Dasumia crassipalpis*. Another very important issue is that the arrangement of cheliceral teeth in *Dasumia mariandyna* clearly does not conform with the characteristic arrangement of cheliceral teeth in this genus. Nevertheless, *Dasumia mariandyna* justlike *Dasumia gasparoi* sp. n. and *Dasumia crassipalpis*, also possesses 3 claws on tarsi III and IV. In accordance with the data mentioned above and by considering embolus/bulbus proportion of the species, the place of *Dasumia mariandyna* in the subfamily Harpacteinae should be rediscussed, for it is possible that *Dasumia mariandyna* may belong to another genus.

**Table 4.** Leg spination of *Dasumia crassipalpis*

♂	**Leg I**	**Leg II**	**Leg III**	**Leg IV**
**C**	0	0	1 pl 1 D	7 pl 2 D
**Tr**	0	0	0	1 rl
**Fe**	4 pl	1 pl	3 D 3rl	9 D
**Pa**	0	0	2 D 1 rl	1 D
**Ti**	0	0	2 pl 1 D 3 rl 5 V	4 pl 1 D 3 rl 5 V
**Me**	0	0	3 pl 6 rl 2 V	5 pl 5 rl 5 V

## Results and discussion

With the description of*Dasumia gasparoi* sp. n., the total number of *Dasumia* species is now 14 and the total number of dysderid spiders known from Turkey is raised to 47. Even if we ignore *Dasumia sancticedri* Brignoli, 1978 (described in the genus *Dasumia* and associated with *Dasumia crassipalpis* by Brignoli) which has a suspiciously differentpalpal structure questioning its correct placement in the genus *Dasumia* (see Brignoli, 1978, page 173. figures 1, 2); it is not unreasonable to think that spiders exist in the Eastern Mediterranean basin includes similar but different species which are slightly different from the European taxa in the structure of copulatory organs. The relationships between the European and Eastern Mediterranean representatives of the genus will be clarified following future revisions and with studies including molecular systematics.

## Supplementary Material

XML Treatment for Dasumia gasparoi

XML Treatment for Dasumia crassipalpis

XML Treatment for Dasumia mariandyna
